# Selecting the Appropriate Speed for Rotational Elements in Human-Machine Interfaces: A Quantitative Study

**DOI:** 10.16910/jemr.17.1.1

**Published:** 2024-01-29

**Authors:** Mu Tong, Shanguang Chen, Wenzhe Tang, Yu Zhang, Chengqi Xue

**Affiliations:** School of Mechanical Engineering, Southeast University, China

**Keywords:** Just Noticeable Difference In Speed, Motion Perception, Human-Machine Interface, Perceived speed, Threshold Measurement

## Abstract

The motion of rotation, which served as a dynamic symbol within human-computer interfaces, has garnered extensive attention in interface and graphic design. This study aimed to establish speed benchmarks for interface design by exploring visual system preferences for the perception of both simple and complex rotating icons within the velocity range of 5-1800 degrees per second. The research conducted two experiments with 12 participants to examine the observers’ just noticeable difference in speed (JNDS) and perceived speed for rotational icons. Experiment one measured the JNDS over eight-speed levels using a constant stimulus method, achieving a range of 14.9-29%. Building on this, experiment two proposed a sequence of speeds within the given range and used a rating scale method to assess observers ' subjective perception of the speed series' rapidity. The findings indicated that speed increases impacted the ability to differentiate between speeds; key points for categorizing low, medium, and high speeds were identified at 10, 180, and 720 degrees/s, respectively. Shape complexity was found to modulate the visual system's perception of actual speed, such that at rotation speeds above 180 degrees/s, complex icons appeared to rotate faster than simpler ones. Most importantly, the study applied quantitative methods and metrology to interface design, offering a more scientific approach to the design workflow.

## Introduction

**Figure 1.**
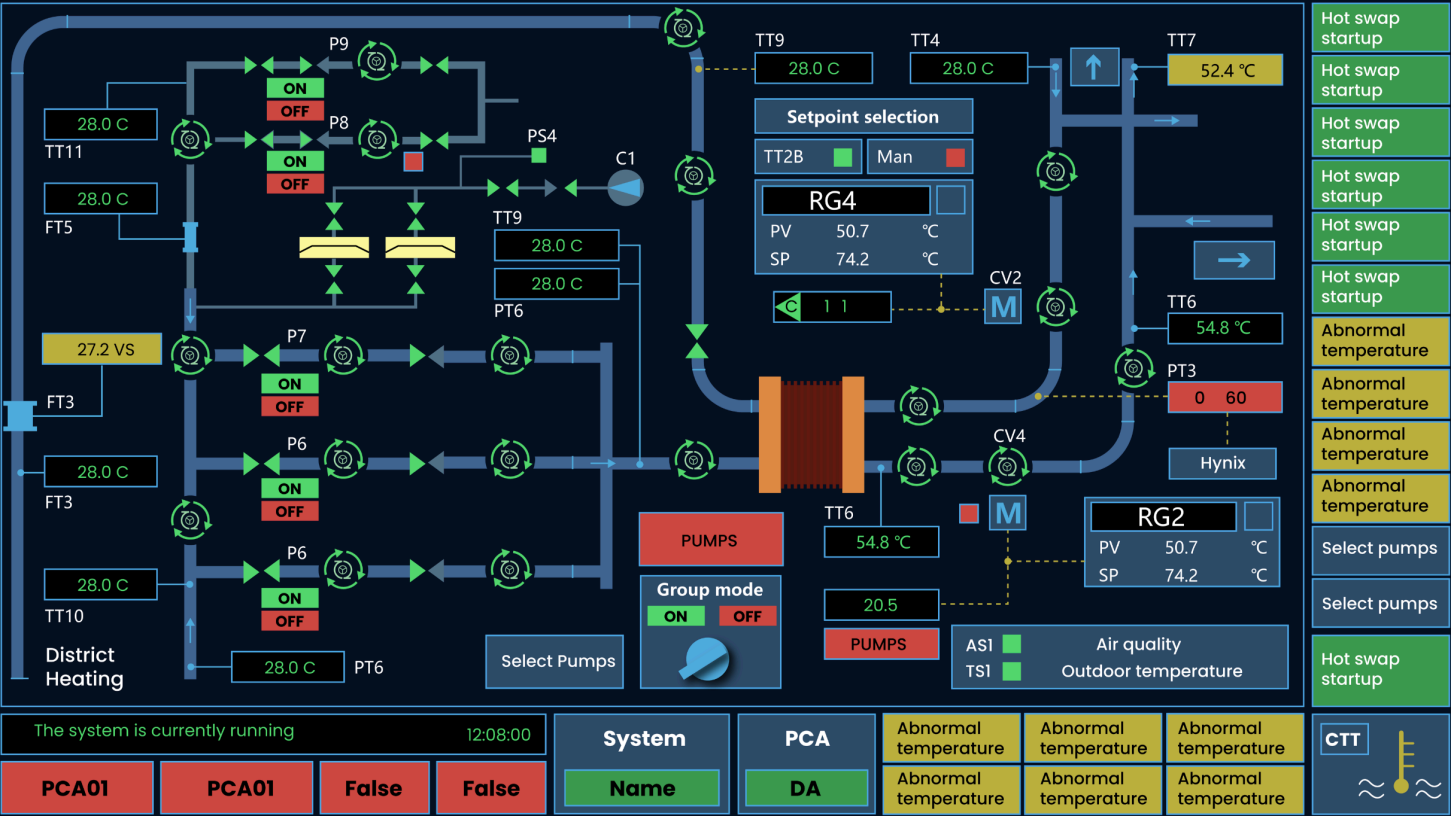

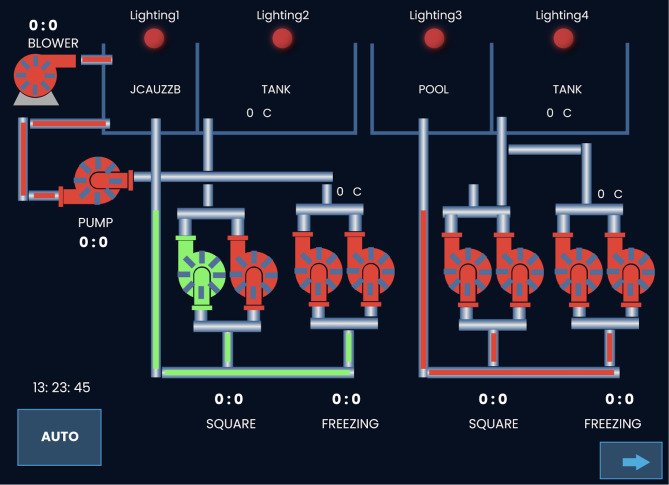
The Application of Rotating Elements in Human-Computer Interfaces (The rotation symbol is used to indicate the speed of water and energy flow).

Rotation was commonly used in the Human-Machine Interface (HMI) to convey dynamic process-related information or visualize other categories of information. Especially in situational awareness systems(31) or dynamic maps(12; 13), as shown in Fig. 1.However, a major challenge in designing rotational elements was determining the appropriate rotation speed. Excessive rotation could cause visual discomfort, such as dizziness and fatigue(7; 24), or even lead to the wagon wheel effect(46). On the other hand, if the speed was set too low, it would fail to effectively communicate important temporal information. To address the issue of determining optimal rotation speeds, it is essential to consider three aspects.

First, it is important to understand the visual system's cognitive mechanism for perceiving rotating objects, specifically how the human visual system recognizes rotation speeds. Two viewpoints were widely accepted on this matter. The first posited that the visual system estimated overall rotational speed through the evaluation of the linear speed of the rotating object. The second viewpoint contended that the brain could directly assess and compute the angular speed of the rotating object, despite angular speed being seemingly more challenging to directly obtain. A consensus remained elusive regarding these perspectives. Kaiser measured subjects' speed discrimination capabilities in a cube rotation experiment, suggesting that the human visual system can indeed perceive the overall angular speed of objects(29). This perception appeared comparable to linear speed perception and was influenced by viewing angles and object structure. Werkhoven and Koenderink conducted measurements on the angular speed of rotating points at different centrifugal distances on a disk, suggesting that angular speed cannot be directly estimated. They proposed that the human eye derived angular speed by estimating the tangential speed of the rotating object, as experimental results implied that angular speed discrimination at the same angular speed depended on spatial distance, specifically the distance between moving objects and the center of rotation(60). Some studies also suggested that the visual system can concurrently track both linear and angular velocities. Barraza and Grzywacz held the view that the visual system can perceive both linear and angular velocities, each relying on distinct mechanisms (2). When the signal quality of the rotating object is high, angular speed perception is more accurate, but it significantly declines in cases of low signal quality. Recent research has provided new evidence suggesting that users can make judgments based on angular speed (39). Some researchers demonstrated that contour shape affects judgments of object angular speed by using rotating objects with varying contour curvature features, offering clues supporting the direct assessment of angular speed by the visual system(6). Considering the focus of this study on rotational elements within HMI, which may assume diverse shapes and sizes, employing linear speed for assessment would introduce complexity into calculations and analyses. To facilitate result analysis and application, angular speed was opted as the parameter for the rotational speed of these elements in this study.

Second, in order to select an appropriate rotation speed, it is also necessary to understand the visual system's perception preferences for rotation speeds. Past research in this area had largely focused on measuring the ability to distinguish between different speeds. The Just Noticeable Difference in Speed (JNDS) has garnered widespread attention because it served as an effective indicator for gauging the visual system's sensitivity to speed changes(5; 41). Understanding the limitations of perceptual ability through JNDS could also aid in optimizing the presentation of dynamic images(14). In a rotating cube experiment conducted by Kaiser, values ranging from 8% to 20% were reported for rotation JNDS. These values were influenced by factors such as viewing angles, initial phases, and stimulus sizes(29). Werkhoven and Koenderink's study on rotating annular random dots around a fixed center observed a minimum JNDS value of 7% (60). Another experiment achieved a 5% JNDS with a rotational speed of 75 degrees/s (30). Disparities in measurement outcomes were attributed to stimulus form, texture density, and size, among other factors. However, these studies in the field of psychophysics often employ abstract shapes composed of dynamic dots or spheres as stimuli. While some studies have extended the motion of dots to the perception of more realistic rotating objects(10). However, these objects differ significantly from common rotating objects found in human-machine interfaces (HMI) in terms of shape, size, and texture. These three categories of features have been shown to affect measurements of rotational speed perception(2; 60). Moreover, in the above studies, graphical presentation mediums included projection, oscilloscopes, or direct observation of real objects. Nevertheless, in HMI settings, objects were typically presented on high-resolution screens with a high refresh rate. This difference directly affected contrast levels, which was considered one of the key factors affecting the perception of speed(53, 54). Therefore, to obtain more accurate results, this study reinitiated the measurement of JNDS under the human-computer interface environment.

Finally, to quantitatively solve the issue of setting rotational speeds, it is also essential to establish a reference scale for the rapidity of rotation speeds, thus providing a quantitative reference for selecting appropriate speeds. This requires measurement based on the observer's psychological perception of the actual rotational speed. Compared to the actual speed, the perceived speed served as a psychophysical index that more closely reflected the observer's real sensations and could be directly applied to measure the subjective perception of speed in dynamic objects within the interface. (22; 27; 62). This direct method of measuring human perceptual scales through a rating scale was common in the field of psychophysics for the quantitative study of the intensity of perception brought about by physical stimuli(35; 49; 51). Additionally, to make the conclusions of the study more universally applicable, the complexity of the stimuli was also measured as an independent variable, because past research has shown that the complexity of the stimuli was a critical factor affecting human performance in cognitive tasks(28; 61).

Based on the above, the purpose of this study was to investigate the discriminative ability and perceptual preferences of the human visual system for the rotational speed of different types of stimuli, to select appropriate speeds for rotating objects in interfaces. The study included two experiments. In the first experiment, the observers’ JNDS was measured, obtaining a cognitive rule for speed discrimination within a wide range of speeds, as well as a set of speeds that could be clearly distinguished. In the second experiment, a scale was used to examine the observers' perceived speed of rotation. By dividing the perceived speed into high, medium, and low ranges, an objective measurement scale for rotation speeds within 0-1800 was provided. The ultimate results of the study were beneficial to optimizing the presentation of the rotating elements in interface design, thereby enhancing the overall cognitive efficiency and user experience of the interface.

## Experiment 1 Measurement of JNDS for Rotational Speed

## Methods of Experiment 1

### Design

**Figure 2.**
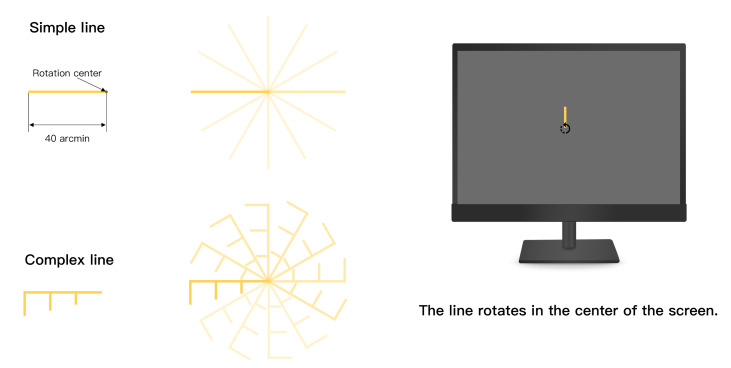
Design of simple and complex line segments, as well as schematic diagrams of rotation on the screen.

Yellow objects spinning in the center of a black background served as the experimental stimulus, and this color combination has been proven to enhance visual sensitivity(32; 55). According to the previous studies on the definition of visual shape complexity, as well as recommendations from expert users(1; 3), single line segments and composite line segments were chosen as the stimuli to represent rotational shapes of varying complexity in the HMI. The latter exhibited greater complexity, characterized by more visual feature points and higher levels of asymmetry compared to the former. Referencing some research suggestions, the length of the line segments in the stimuli was set at 40 arcmin(25; 55), with the end of the line as the rotation center. The stimulus rotated counterclockwise along the vertical axis at a radius equal to its length, as illustrated in Fig. 2. The contrast level was 8.6, screen brightness was 150 cd/m² and environmental illuminance was 300 lux. When selecting the speed range, considerations were made regarding the maximum speed that the human eye can effectively track and the limits to which a screen can accurately display motion(10; 19; 46; 58). This was also informed by speed points commonly examined in past research(6; 30). Guided by the advice of two human-computer interface design experts, the final speed intervals chosen for measurement were 5, 10, 30, 90, 180, 360, 720, and 1800 degrees per second. These selections aimed to cover a sufficiently broad range of speeds while excluding excessively high or low speeds, which lack practical relevance.

### Participant

Participants for this experiment included 12 graduate students (6 males and 6 females) from Southeast University's Institute of Human-Computer Interaction, all of them were between the ages of 22 and 28 (M=25.2, SD=1.35). The sample size was tested using G-power, with the effect size of 0.04 and the power of 0.95. All participants had normal or corrected-to-normal vision and no history of psychiatric disorders or eye-related diseases. Additionally, they all had experience in HMI design. Before participating, they read and signed an informed notification about the experiment, which was authorized by the Ethics Review Committee of the Institute of Human-Computer Interaction at Southeast University under number 20230328001.

### Produce

The thresholds were measured based on previous studies (40; 43) using the constant stimulation method for more accurate results. First, the experimenter introduced experimental procedures, important considerations, and operational methods in the experiment to the participants. The experiment involved two types of stimuli: a standard stimulus and a contrast stimulus. The standard stimuli consisted of rotating shapes at various speeds (5, 10, 30, 90, 180, 360, 720 and 1800 degrees/s).

Prior to the official experiment, a pre-experiment was conducted to familiarize participants with the process and determine comparison stimuli relative to each standard stimulus. The selection of comparison stimuli was carried out in the following three steps. First, the experimenter set predefined intervals for different standard stimuli based on past research, and the arithmetic mean of the upper and lower limits of these intervals was the standard stimulus(20; 29). Second, at each speed condition, the upper and lower limits of the preset interval were changed step by step at fixed intervals, requiring the participants to judge whether the adjusted upper and lower limits were faster or slower than the standard stimuli, with each level requiring 10 judgments. Third, the stimulus was established as the upper and lower limits for comparison stimuli in the formal experiment when the correct judgment rate of the participant dropped below 90%. Furthermore, seven equidistant stimuli within this established range were selected as the comparison stimuli for the corresponding standard stimulus.

**Figure 3.**
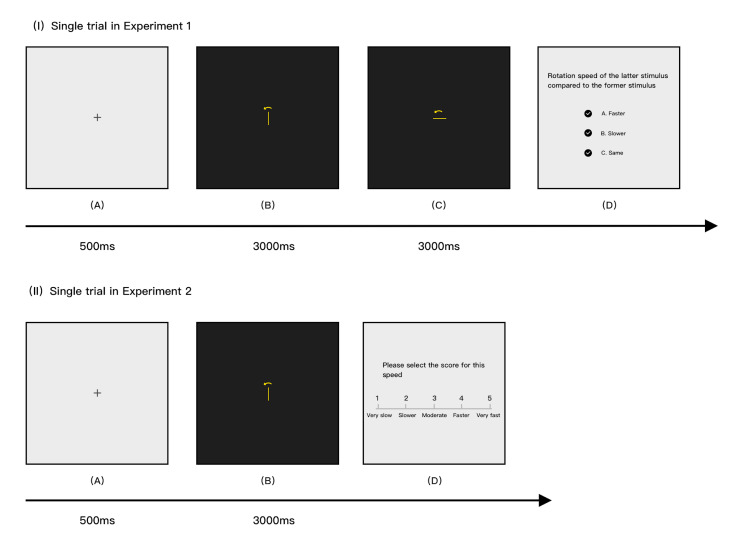
Diagram of the single trial process for two experiments

Before the formal experiment, participants established the upper and lower bounds for the comparison stimuli through the preliminary process. Once this phase concluded, participants advanced to the formal experiment. As illustrated in Figure 3, the formal experiment required participants to observe two stimuli that appeared consecutively on the screen, one being the standard stimulus and the other being the comparison stimulus. Each was displayed on the screen for 3000ms. Participants then evaluated the speed of the two stimuli in the following interface, choosing "+" for faster, "-" for slower, and "=" for equal speed. This evaluation was repeated 40 times, with complex lines and simple lines each repeated 20 times. Additionally, the presentation sequence of the comparison and standard stimuli was randomized using a counterbalancing method. The total experiment comprised eight blocks, each with 280 trials (7 comparison stimuli repeated 40 times). Participants could take breaks at their discretion within these blocks to prevent fatigue. To minimize sequence effects, the order of the blocks was randomized, and a rest period exceeding 10 minutes was mandated between blocks. The experiment continued only after participants verbally confirmed they were ready to proceed without significant fatigue.

### Apparatus and Environment

The program for the formal experiment was built on E-prime 3.0, and the dynamic stimulus material was converted into the dynamic video using Adobe Effect 2020 and presented through E-prime’s video module. The program was run on an HP workstation with a CPU frequency of 2.4GHz and a software system environment of Windows 10. The display screen was a 27-inch monitor with a resolution of 1920*1080 and a refresh rate of 120Hz. Screen brightness could be adjusted, reaching a maximum of 300 cd/m2. To ensure participants' line of sight was centered on the screen, they were seated on a height-adjustable chair positioned at a distance of 510mm from the screen, which was a commonly used distance in the VDT work environment(37; 50; 56). The measurement of indoor illuminance and screen brightness followed the guidelines outlined in GB/T 5700-2008. The test area was measured using the four-corner distribution method to calculate an average illuminance value. A luminance meter placed at the height of the subject's eyes measured screen brightness three times to obtain an average value of the screen brightness. The luminance meter used was manufactured by XinBao Scientific Instruments, with model number SM208. The equipment used for measuring indoor illuminance has model number DL-333215.

## Results of Experiment 1

**Figure 4.**
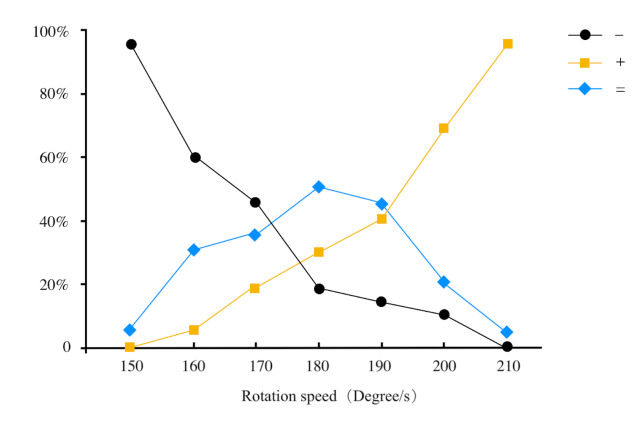
Calculation of JNDS by the method of linear interpolation (when the speed was 180 degrees/s)

A total of 12 valid data sets were collected in the experiment. The data were sorted and the participants' judgment results for the contrasting stimuli were counted under various standard speed conditions. The results were classified and described using the symbols '+', '-', and '='. The statistical data were then plotted using the method of linear interpolation, as shown in Fig. 4. The x-axis of the graph represents the comparison stimulus corresponding to the standard stimulus, while the y-axis represents the percentage of judgments. A reference line at 50% was used, and formula (1) was applied to calculate the upper and lower thresholds for this speed.).

**(1):**
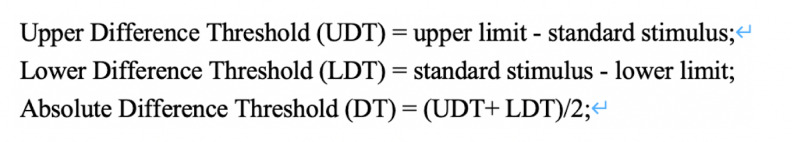


**Figure 5.**
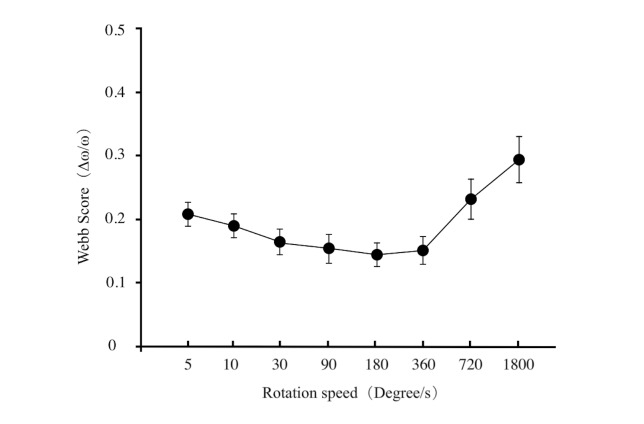
The Weber scores line chart expressed the trend of JNDS changes with speed.

Descriptive statistics for all rotation JNDS are presented in Table 1. A repeated measures ANOVA analysis was performed based on rotation speed and stimulus type. The results in Table 2 illuminate that there is a significant main effect of rotation speed on JNDS(F = 271.81, p < 0.01), while contour complexity had no significant main effect on the JNDS (F = 3.92, p > 0.05), and the interaction effect between the speed and contour complexity was also not significant (F = 0.918, p > 0.05). The results above suggest that the visual system's JNDS is affected by the speed itself, whereas contour complexity had no impact on JNDS. To further analyze the relationship between JNDS and the moving speed, Weber parameters of the difference thresholds were calculated for each standard stimulus, and the relationship graph between the Weber parameter and the speed was plotted in Fig. 5. From this graph, it can be observed that as rotation speed increases, Weber parameters initially decrease and then increase. The minimum value of 0.149 is reached at a rotation speed of 180 degrees/s, while the maximum value of 0.290 is reached at a rotation speed of 1800 degrees/s..

**Table 1.** Descriptive statistical results for the JNDS of two types of stimuli

	Simple Stimuli			Complex Stimuli		
Speed	JNDS（M）	JNDS（SD）	Weber fraction	JNDS（M）	JNDS（SD）	Weber fraction
5	0.99	0.06	0.198	1.07	0.07	0.214
10	2.03	0.21	0.203	1.87	0.13	0.187
30	5.25	0.35	0.175	4.65	0.28	0.155
90	12.78	1.78	0.142	14.76	1.74	0.164
180	25.74	2.42	0.143	26.82	3.13	0.149
360	54.00	4.63	0.150	54.72	5.31	0.152
720	167.04	22.58	0.232	158.40	25.02	0.220
1800	511.20	89.75	0.284	550.80	98.55	0.306

Note. The Weber fraction is the ratio of the JNDS average to standard speed. Note. The unit of speed is degrees/s.

## Discussion of Experiment 1

Experiment 1 measured the rotation JNDS for two types of stimuli: simple and complex, within a range of speeds from 5 to 1800 degrees per second. The measurement results for Weber fractions ranged from 14.6% to 29%, indicating that participants performed worse compared to previous studies. (20; 29). This could be attributed to three main factors. Firstly, the type of stimulus could affect the participants' judgment of speed(8, 9). The previous studies used random dots or rotating cubes as stimuli, while this experiment used line segments, which was significantly different.

Observing differences in objects leads to varying cognitive loads, which in turn affects eye movement behavior, particularly micro-saccades(4; 34; 44). This inconsistency in eye movements may have led to an uneven distribution of cognitive resources, which was due to the limited availability of the resources. Specifically, this increased the cognitive load for participants engaged in speed tracking, resulting in fewer resources available for speed recognition.

Secondly, the physical conditions of the display device, such as imaging technology and screen material, particularly contrast of brightness(42; 52), had been shown to affect participants' judgments of JNDS. Higher brightness and contrast levels had been found to enhance participants' ability to discern speed differences(15; 36). Finally, it has been confirmed that individual factors such as age significantly affect the measurement results of JNDS(38; 47). The age distribution of the participants selected in this study was inconsistent with the above research.

The overall results indicate that human speed discrimination shows variability between fast and slow speeds. Within the speed range set in this experiment, when the rotation speed was less than 180 degrees/s, the Weber value decreased with increasing speed, demonstrating the enhancement in participants' ability to discriminate speeds with higher velocities. As the speed continued to increase beyond 360 degrees/s, the subjects' JNDS gained rapidly until the speed reached 1800 degrees/s, and the Weber value was 29% suggesting a significant weakening of participants' ability to discriminate speeds at the high speed of 1800 degrees/s. This is consistent with previous research showing that when presented with rates close to threshold boundaries, the ability to discriminate speeds decreases rapidly (40; 45). It is speculated that this may be due to the occurrence of motion blur caused by excessively fast rotation speeds(46), which impairs visual perception of speed on the screen and weakens speed recognition abilities(10), ultimately resulting in a significant increase in JNDS.

Experiment 1 measured the speed discrimination thresholds for two types of stimuli in a human-machine interface context. Referring to these thresholds, a set of speed sequences that could be distinguished significantly were selected within the range of 0-1800 degrees/s. The sequence included speeds of 5, 10, 30, 60, 90, 180, 360, 720, 1440, and 1800 degrees/s which covers the typical speeds of rotating icons in the interface environment. Experiment 2 focused on this range of speeds to assess participants' perceptions of actual rotational speeds.

## Experiment 2 Measurement of Perceived Speed

## Methods of Experiment 2

### Design

The experimental stimulus design remained unchanged from Experiment 1. The speed level was set at 10 levels, ranging from 5 to 1800 degrees/s in increments of 5, 10, 30, 60, 90, 180, 360,720,1440 and 1800 degrees/s.

### Participant

The 12 subjects who were recruited for Experiment 1 also participated in Experiment 2. The sample size was tested using G-power, with the effect size of 0.04 and the power of 0.80. The research plan for Experiment 2 was approved by the Ethics Review Committee of the Human-Computer Interaction Institute at Southeast University, with reference number 20230420001.

### Produce

Firstly, the experimenter introduced the procedure, operation methods, and precautions to the participants. Before starting the formal experiment, a practice session was set with 15 practice trials. Participants could increase the number of practice trials until they confirmed their understanding of the procedures verbally. The process of a single trial is shown in Fig. 2. During each trial, a white crosshair appeared at the center of a black background on the screen, and participants were instructed to focus on this point. Subsequently, a changing stimulus was displayed for 3000ms. In the following interface, participants used the mouse to select the perception scale for the stimulus from a 5-point scale. Once completed, participants proceeded to the next trial. The experiment consisted of two blocks based on the complexity levels of the contours. Stimuli within each block were presented in a random order, with a 10-minute break between the two blocks. The two blocks were presented in a counterbalanced manner. Each speed level requires five repetitions for a total of 5*2*10=100 trials per participant. The entire experiment took approximately 40 minutes to complete.

### Apparatus and Environment

The experimental program was written through C# and run on the Unity 2022 platform. The experiment was conducted on an HP workstation with a CPU frequency of 2.4GHz and a software system environment of Windows 10. The display screen size was 27 inches with a resolution of 1920*1080 and a refresh rate of 120 Hz. The maximum brightness level for the screen was set at 300 cd/m² and could be adjusted accordingly. Participants were requested to sit on an adjustable chair positioned 510mm away from the screen to ensure that their line of sight was at the center of the screen. The brightness settings for both the screen and environment followed those used in Experiment 1

## Results of Experiment 2

**Table 2.** Descriptive Statistical Results of Experiment 2

Perception Scale		
Speed（degrees/s）	Average value（M）	Standard deviation（S）
5	1.00	0.00
10	1.24	0.22
30	1.84	0.30
60	2.18	0.22
90	2.23	0.30
180	2.79	0.43
360	3.28	0.34
720	4.28	0.51
1440	4.81	0.32
1800	4.96	0.19

**Figure 6.**
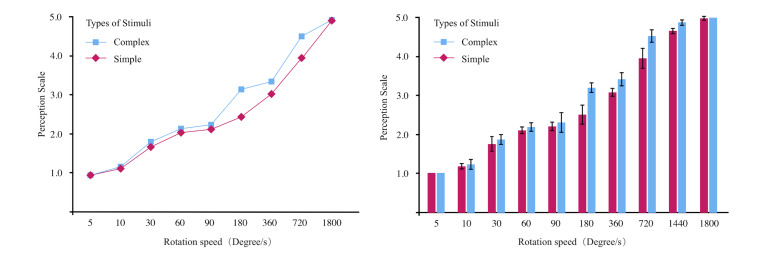
The line graph and mean plot describe how the perceived scale scores change with speed.

A total of 1200 data points were collected in this experiment. Descriptive statistics and variance analysis were performed on the experimental data using SPSS. Before the analysis, scatter plots were created for perception scale and speed, and obvious outliers were manually removed, resulting in the exclusion of 7 discrepant data points. The data were then standardized and no abnormal values were found. The descriptive statistical results of the experiment are shown in Table 2, and a line graph depicting the mean perception scale values according to the stimulus type was illustrated in Fig. 6. The results indexed that participants' perception scale consistently gained with an increase in rotation speed. After the rotation speed exceeded 180 degrees/s, significant differences between mean values for both types of stimuli began to emerge. When the speed reached 1800 degrees/s, the perception scale for both types of stimuli approached their highest value with minimal difference.

A repeated measures ANOVA analysis was performed on the data, and variance analysis results showed that both the main effect of stimulus type and rotation speed on the perception scale were significant (F=26.65, p<0.01; F=387.3, p<0.01), as shown in Table 3. There was a significant interaction effect between stimulus type and rotation speed (F=107.28, p<0.01). The simple effect analysis of the interaction between the rotation speed and shape complexity proved that when the rotation speeds were at 180, 360, 720, and 1440 degrees/s, the scales corresponding to complex shapes were significantly higher than those of simple shapes (p <0.01). When rotation speeds were below 180 degrees/s and at 1800 degrees/s, there was no significant difference between the two types of stimuli in perceived scales (p >0.05). In addition, a post-comparison of rotation speed based on the LSD method showed that when the rotation speed was greater than 10 degrees/s but less than 1800 degrees/s, a higher speed always contributed to a higher perceived scale (p<0.001). These results pointed out that both the shape complexity and the speed had a bearing on the visual system's perception for observing rotational stimulus.

**Table 3.** Results of the variance analysis for the two experiments

Variant	Experiment 2		Experiment 2	
	F	P	F	P
Rotation speed	271.81	0.00	387.30	0.00
Stimulus type	3.92	0.06	26.65	0.02
Interaction	0.913	0.32	107.28	0.00

## Discussion of Experiment 2

In Experiment 2, the subjective perception scale of rotation speed was measured for a given set of speed sequences. Overall, Enhancements in rotation speeds led to higher perceived scores for speed, which aligned with previous research (11; 52). The measurement results also demonstrated that shape complexity affects the judgment of the perceptual scale. Specifically, when the speed exceeded 180 degrees/s, complex-shaped stimuli resulted in higher perceived speed scales compared to simple-shaped stimuli. This could be attributed to the multi-feature points and the more asymmetric complex contour led to greater optical flow changes during the rotation process, thereby impacting the visual system's judgments of optical flow speed(18; 33). However, once the speed reached above 1440 degrees/s, there was no significant change in human visual perception of rotational speed. Both types of rotational stimuli were consistently perceived as very fast with a score of 4.80. These findings provide preliminary definitions for rotation speeds within an HMI environment. The rotation speed below 180 degrees/s can be defined as slow; the rotation speed below 10 degrees/s can be defined as very slow; and the rotation speed between 180 degrees/s and 720 degrees/s can be defined as fast. When the speed exceeds 720 degrees/s, it can be defined as extremely fast.

## General Discussion

To improve the usability and user experience of dynamic HMI, designers tend to adopt a more scientific approach rather than rely on intuition when designing dynamic elements. This study focuses on rotating icons in dynamic HMI and measures people's speed discrimination ability and subjective preferences for rotating icons through two experiments. Experiment 1 reveals that as the speed of rotation increases, the discrimination threshold initially decreases before eventually increasing. These findings were consistent with previous measurements (40; 45), indicating that the visual system exhibited similar trends in distinguishing the speed of different types of motion. Experiment 2 measured the perceived scale for a given speed. It was found that the perceived scale of speed increased as the actual speed increased until reaching a threshold at 1440 degrees/s. In addition to obtaining specific data about the speed perception of rotating objects through measurements, the two experiments also discovered some new findings. Apart from obtaining specific data regarding the speed perception of rotating objects through these measurements, both experiments also yielded some new findings.

Firstly, there were significant differences in JNDS between fast and slow speeds. Previous research suggests that these differences can be attributed to the different systems involved in processing fast and slow motion in visual pathways. The ventral pathway (V3, V4) was believed to process slow-speed motion, while the dorsal pathway (MT) processes fast-speed motion in the human brain(21; 26, p. 18). This could also be explained by the separate speed processing mechanism for slow-speed and fast-speed optic flow patterns(17; 57), where faster optic flow velocities activated more systems(57) or neurons(16) to enhance speed processing and calculation. Secondly, research has found that shape affects participants' perception of rotation speed but does not affect their discrimination of speed differences. This may sound confusing, but two reasons can explain it. Firstly, the processing of visual information for shape and speed is independent and occurs through separate pathways known as the P pathway and M pathway(21; 48; 59). Therefore, shape does not directly influence the visual system's processing of speed, which explains why shape does not affect the discrimination of speed differences.

However, due to limited cognitive resource allocation, in the speed discrimination experiment, the emphasis was placed on the importance of discriminating speeds, which forced users to invest numerous resources into judging speed while disregarding specific details about the rotating object's shape. On the other hand, in the subjective perceptual experiment, participants are only instructed to judge speed based on their understanding. Apart from perceiving speed, they also allocate some attention to clearly discerning the contours of shapes. When presented with complex shapes at identical speeds, more cognitive resources are occupied by perceiving clear outlines(23), which results in fewer resources available for perceiving speed accurately and thus affecting perceived rates.

## Conclusion

The current study provided recommendations and reference benchmarks for setting the rotation speed of objects in an interface environment by measuring observers' perceptual preferences for rotational speed. Overall, participants' ability to discern speed showed a trend of initially rising and then declining as the speed advanced, with the best performance observed in the moderate speed range. difference threshold could reach a level of 15% when the rotation speed was at 180 degrees/s. Furthermore, it was found that the complexity of rotating stimuli influenced participants' subjective perception of speed. More complex shapes appeared to rotate faster at the same rotational speed. This suggests that complex icons or rotating stimuli should be redesigned or simplified for better visual communication in HMI design. Based on the measurement results, three speeds (10 degrees/s, 180 degrees/s, and 720 degrees/s) can be used as boundaries to categorize low-speed, medium-speed, and high-speed rotations in HMI objects. However, when applying these classifications in specific applications, other factors within HMI such as stimulus size, color combinations, and additional indicators need to be considered comprehensively.

### Limitation

This study has some limitations that should be further considered in future research. It should be noted that since age can have an impact on performance in dynamic cognitive and speed perception tasks, this study did not conduct relevant measurements and distinctions among different age groups. This may lead to the final measurement conclusions of the article not applying to the elderly population. It is recommended that future research measures the speed perception and discrimination capabilities of different groups within dynamic interface environments.

### Ethics and Conflict of Interest

The author(s) declare(s) that the contents of the article are in agreement with the ethics described in http://biblio.unibe.ch/portale/elibrary/BOP/jemr/ethics.html and that there is no conflict of interest regarding the publication of this paper.

### Acknowledge

This study was partly supported by the National Natural Science Foundation of China (grant no. 72271053).

This study was also partly supported by the China Scholarship Council scholarship (grant no. 202206090270).
